# A Pilot Study of the Effect of Sodium Thiosulfate on Urinary Lithogenicity and Associated Metabolic Acid Load in Non-Stone Formers and Stone Formers with Hypercalciuria

**DOI:** 10.1371/journal.pone.0060380

**Published:** 2013-04-08

**Authors:** Onyeka W. Okonkwo, Ruchika Batwara, Ignacio Granja, John R. Asplin, David S. Goldfarb

**Affiliations:** 1 Department of Medicine, New York Harbor VA Healthcare System and NYU School of Medicine, New York, New York, United States of America; 2 Nephrology, Newark Beth Israel Medical Center, Newark, New Jersey, United States of America; 3 Litholink Corp, Chicago, Illinois, United States of America; 4 Nephrology Section, New York Harbor VA Healthcare System, and NYU Langone Medical Center, New York, New York, United States of America; Universidade de São Paulo, Brazil

## Abstract

**Background and Objectives:**

Sodium thiosulfate (STS) reduced calcium stone formation in both humans and genetic hypercalciuric stone forming (GHS) rats. We sought to measure urine chemistry changes resulting from STS administration in people.

**Design, Setting, Participants & Measurements:**

STS was given to healthy and hypercalciuric stone forming adults. Five normal non-stone forming adults (mean age 33 years), and 5 people with idiopathic hypercalciuria and calcium kidney stones (mean age 66 years) participated. Two baseline 24-hour urine collections were performed on days 2 and 3 of 3 days of self-selected diets. Subjects then drank STS 10 mmol twice a day for 7 days and did urine collections while repeating the self-selected diet. Results were compared by non-parametric Wilcoxon signed rank test. The primary outcome was the resulting change in urine chemistry.

**Results:**

STS administration did not cause a significant change in urinary calcium excretion in either group. In both groups, 24 hour urinary ammonium (P = 0.005) and sulfate excretion (P = 0.007) increased, and urinary pH fell (P = 0.005); citrate excretion fell (P<0.05) in hypercalciuric participants but not in non-stone formers. Among stone formers with hypercalciuria, 3 of 5 patients had measurement of serum HCO_3_ concentration after the STS period: it did not change. The net effect was an increase in supersaturation of uric acid, and no change in supersaturation of calcium oxalate or calcium phosphate.

**Conclusions:**

The basis for studies demonstrating that STS prevented stones in rats and people was not reflected by the changes in urine chemistry reported here. Although serum HCO_3_ did not change, urine tests suggested an acid load in both non-stone forming and hypercalciuric stone-forming participants. The long term safety of STS needs to be determined before the drug can be tested in humans for long-term prevention of stone recurrence.

## Introduction

Sodium thiosulfate (STS) has a number of uses in medicine, the most well known of which is its role as an antidote for cyanide poisoning. It acts via the conversion of cyanide to the more water-soluble thiocyanate which is readily excreted in urine [1]. STS is also commonly administered in conjunction with cisplatin to decrease the incidence of associated nephrotoxicity by functioning as an antioxidant [2]. It is apparently useful in the management of calciphylaxis, or calcific uremic arteriolopathy [3], [4].

Two studies, one in genetic hypercalciuric stone-forming (GHS) rats and one in humans, have shown a decrease in the incidence of kidney stones following STS administration [5], [6]. However the mechanisms by which STS accomplishes this effect remain unclear. Despite reduced stone formation, GHS rats given STS showed increased urine calcium excretion. Urine chemistry also suggested net acid production, raising concern that STS, if given over extended periods of time, may be associated with sustained metabolic acidosis and loss of bone mineral density (BMD). With the increasing prevalence of nephrolithiasis over the last decades [7], the significant morbidity and healthcare costs associated with the management of this condition, and the need for additional preventative modalities, the potential efficacy of STS for the prevention of recurrent nephrolithiasis merits investigation.

Our study sought to investigate whether urine chemistries of humans on STS therapy would suggest efficacy in reducing urinary lithogenicity, and whether evidence for an associated acid load would be present.

## Methods

### Patient Population

**Figure 1:**
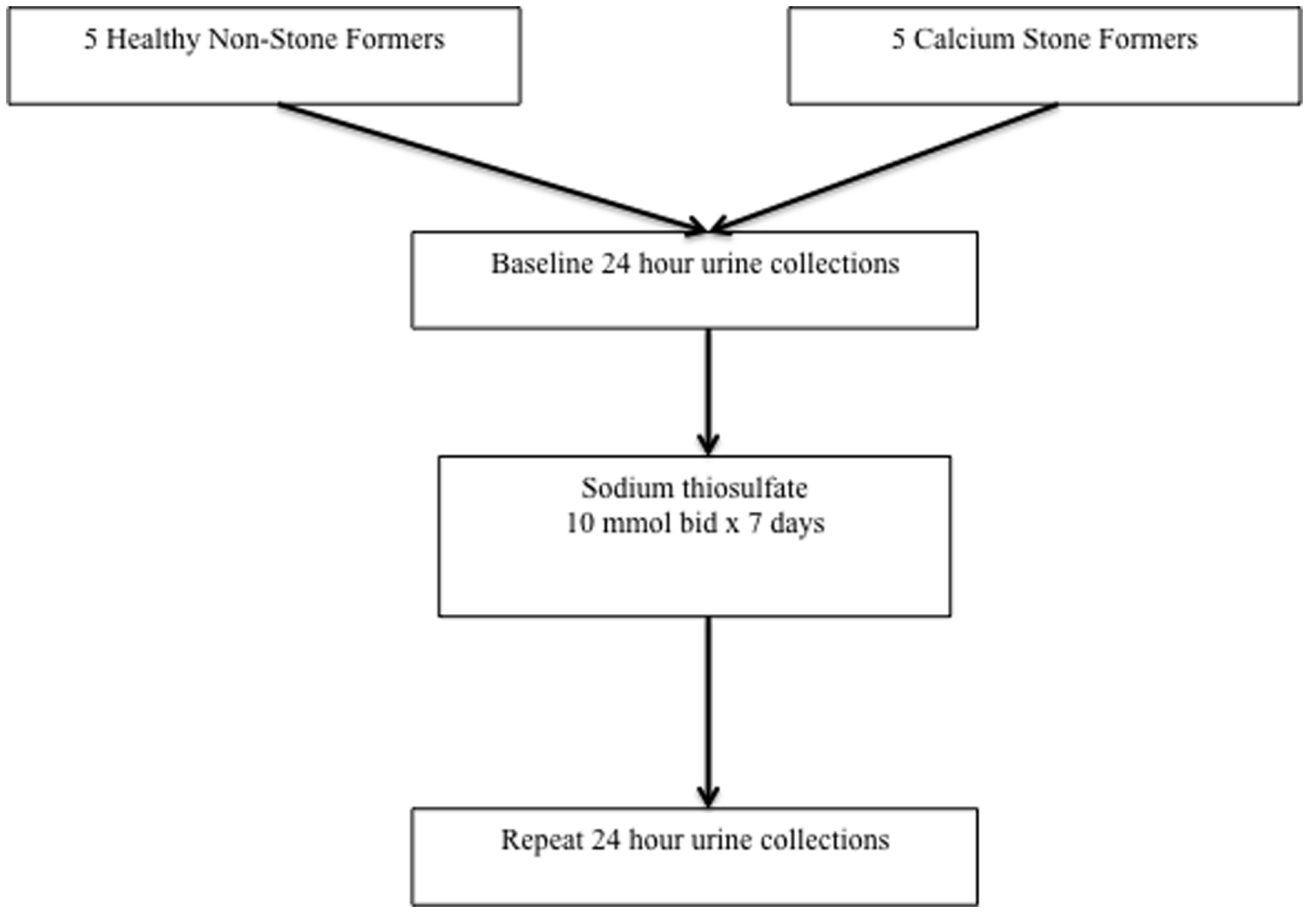
Patient Flowchart and Experimental Design.

The study population consisted of two distinct arms. Figure 1 depicts an outline of the patient flowchart and experimental design. As this was a pilot study, the small sample size was considered adequate to demonstrate whether the dose would lead to clear changes in urine chemistry that could later be used to estimate an effect size. The first group consisted of 5 participants (3 men and 2 women) with a mean age of 33 years old who were without medical co-morbidities, and no history of urolithiasis. The participants were healthy fellow healthcare workers, randomly approached by the authors, who indicated a willingness to participate in a study which included administration of a drug and required multiple urine collections. The participants were recruited between January-March, 2010.

The second group consisted of an equal number of participants with a mean age of 66 years. This group included 4 men and 1 woman all of whom had been seen and evaluated as outpatients in the Metabolic Stone Clinic and who were documented calcium stone formers with a history of hypercalciuria, specifically with 24 hour urine calcium measurements of greater than 300 mg/d on collections done within a year prior to the study, while following a self-selected diet. We identified 10 eligible patients seen in the Metabolic Stone Clinic in recent months and the first 5 who were contacted and agreed to participate were enrolled. The participants were recruited between January-April, 2011. The mean serum creatinine concentration was 1.1+0.1 mg/dl (range 0.9–1.2 mg/dl) and the mean estimated glomerular filtration rate was 74.2+12.4 ml/min/1.73 m^2^ (range 60.0- 91.0 ml/min/1.73 m^2^). Three patients had calcium stones composed of calcium oxalate and calcium phosphate, the latter component constituting 20, 40, or 50% of analyzed stones. The other 2 patients did not provide stones for analysis but had radio-opaque stones seen on plain abdominal radiography. Patients over the age of 80 were excluded from the study. Patients who had hypercalciuria in the past but did not have hypercalciuria on baseline collections done immediately prior to STS administration were not excluded. All patients were prescribed a diet demonstrated in a randomized controlled trial to reduce the incidence of recurrent stones in participants with hypercalciuria (more dietary calcium, less animal protein, sodium and oxalate). [8] Adherence to the dietary measures was encouraged and not monitored. Medications prescribed for kidney stone prevention were prohibited during the urine collections and were to be stopped 2 weeks before the baseline collections were done. Two patients had been prescribed thiazides: one was taking chlorthalidone 25 mg once a day and one was taking hydrochlorothiazide 25 mg once a day. No patients had been prescribed potassium citrate.

The patients were selected at random and solicited for participation. The study was approved by the institutional review board of the New York Harbor VA Healthcare System and registered with Clinicaltrials.gov as NCT01088555. The study was performed under US Food and Drug Administration Investigational New Drug number 106,424. All subjects provided written consent to participate in the study.

### STS Administration and Urine Collections

Each participant undertook 2 baseline 24 hour urine collections over two consecutive days during which they were instructed to keep detailed food diaries while eating their self-selected diets. Oral doses of STS were then taken by the subjects for 7 days after the completion of the baseline urine collections. STS was dispensed by the research pharmacist to the patients and taken home for self-adminstration. The dose was 10 mmol (5 ml of a 2 M solution) twice a day, replicating the protocol described by Yatzidis. The taste and smell of STS precludes blinding the study. On days 5, 6 and 7, subjects replicated the diets they had documented during the pre-STS baseline urine collections. On days 6 and 7 of the medication administration period, participants undertook a second set of two 24 hour urine collections. Food diaries were not collected; they were simply used to allow subjects to replicate diets. Three of the 5 stone forming participants also had basic metabolic panels, including serum bicarbonate levels, checked prior to STS administration and on day 7 of taking STS; the other 2 did not have post-STS serum measurements because of logistical problems.

### Urine Chemistry Analysis

During the urine collections, the urine was maintained at room temperature. An antimicrobial and a urine volume marker were added to each urine container and then a 50 ml aliquot of urine was obtained. The participants performed the urine collections at home, and then mailed their urine collections to Litholink Corp (Chicago, IL) for analysis [9].

In each 24 hour urine sample, we measured calcium, chloride, creatinine, magnesium, sodium, potassium, phosphate, ammonium, and uric acid concentrations by standard laboratory technique using a Beckman Synchron CX5 (Beckman Instruments, Brea, CA, USA). pH was measured by glass electrode. Oxalate was measured by enzyme assay using oxalate oxidase (Trinity Biotech, Bray, Ireland). Citrate was measured by enzyme assay using citrate lyase (Mannheim Bohringer, Mannheim, Germany). From these analyses supersaturation (SS) was calculated with respect to calcium oxalate, calcium phosphate, and uric acid using the iterative computer program EQUIL 2 [10]. For each of the 3-day experimental phases the mean values of the two 24 hour collections were reported. The results of one individual in whom 24 hour excretion of creatinine varied between collections by 30% or more were judged to have been inaccurately collected and 1 of 2 samples was excluded.

Thiosulfate and sulfate were measured by ion chromatography using a Dionex ICS 2000 system (Dionex Corp., Sunnyvale, CA). Samples were loaded into a 25-µl loop using an autosampler and injected onto an AG-11 guard column and AS-11 analytical column in series, with KOH as the mobile phase. Ion peaks were detected using a conductivity meter with the eluent background conductivity suppressed using an anion self-regenerating suppressor. We have found thiosulfate to be stable in urine under the collection conditions used in this study.

### Statistical Analysis

Results of baseline and post-STS urine collections were compared by non- parametric Wilcoxon signed rank test and the effects of STS in controls and hypercalciuric stone forming participants were compared by Mann Whitney U test. Statistical data were generated and analyzed with a commercially available software package, Systat (Point Richmond, CA, USA). Results were expressed as mean±SD and differences were considered statistically significant at *P*<0.05.

## Results

**Figure 2:**
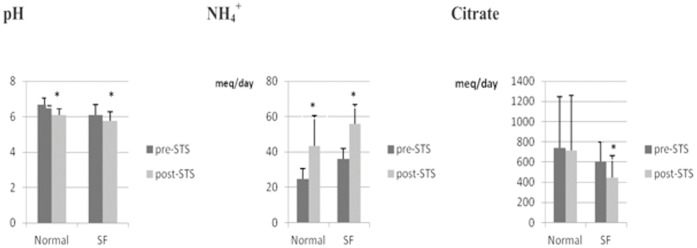
Comparison of pre- and post-STS urine pH, ammonium and citrate excretion in normal (n = 5) and hypercalciuric (n = 5) stone formers (*: P<0.05 post-STS compared with pre-STS).

All 10 patients enrolled completed the study without any lost to follow-up. The results of all participants were included. The results of STS administration in both the normal participants, the hypercalciuric participants and the 2 groups combined are presented in Table 1. There was no change in urinary calcium excretion in the control group or in the hypercalciuric stone formers, though in the former group, a small increase that was not statistically significant occurred (P = 0.08). Figure 2 highlights that in both healthy participants and stone formers, STS administration was associated with increases in urinary ammonium and concomitant decreases in urine pH. Urine citrate excretion did not change in controls but fell significantly in the stone-formers (p<0.05 by Mann-Whitney U test). This was the only effect of STS that differed between normal controls and hypercalciuric stone formers; when the 2 groups were combined, the result was statistically significant (P = 0.03). Urine sulfate excretion rose in both groups but was statistically significant only in the stone-formers (P<0.05) due to a large standard deviation in the control group (P = 0.08). The effect was highly significant when the groups were combined (P = 0.007). However in both groups the absolute amount of sulfate excreted was approximately equal, on a molar basis, to the thiosulfate administered by mouth. On average, less than 5% of the administered thiosulfate dose was excreted in the urine (mean 0.60 mmoles/d, range 0.33 to 1.22 mmoles/d). The net effect was an increase in uric acid supersaturation in both groups and in the combined data, due to the decreased urine pH. A small decrease in supersaturation of calcium phosphate was not statistically significant in either group or in the combined data (P = 0.09). Calcium oxalate supersaturation did not change.

**Table 1:** Effects of STS on 24 hour urine chemistry in hypercalciuric calcium stone formers (n = 5) compared to its effects in normal patients (n = 5), and the 2 groups combined (n = 10); mean±SD.

NORMAL CONTROLS				HYPERCALCIURIC STONE FORMERS			COMBINED		
	Baseline	Post-STS	P-value	Baseline	Post-STS	P-value	Baseline	Post-STS	P value
Ca (mg)	111.5±24.9	147±43.9	0.08	279.3±155.6	277.9±143.9	0.89	195.4±137.4	212.5±121.7	0.17
Na (meq)	186.6±66.8	198.1±61.4	0.5	172.1±46.4	198.8±48.4	0.22	179.4±54.8	198.4±52.1	0.20
K (meq)	97.2±25.0	82.9±22.0	0.22	69.8±15.8	66.0±18.8	0.69	83.5±30.7	74.4±21.3	0.24
Creatinine (mg)	1171.3±377.2	1235.8±422.1	0.14	1748.8±255.7	1858.2±378.0	0.14	1460.1±430.0	1547.0±500.3	0.04
pH	6.67±0.39	6.08±0.40	<0.05	6.09±0.58	5.76±0.52	<0.05	6.39±0.56	5.95±0.48	0.005
NH_4_ (meq)	24.8±5.9	43.4±17.3	<0.05	36.3±6.1	55.8±11.2	<0.05	31.6±7.6	49.6±15.2	0.005
Citrate (mg)	740.6±504.7	713.9±548.6	0.5	605.9±195.1	444.0±221.1	<0.05*	673.3±367.7	579.0±419.2	0.03
PO_4_ (mg)	805.±410	831±496	0.5	1152±297	1033±263	0.08	979±384	932±390	0.57
SO_4_ (meq)	44.1±18.8	85.8±29.3	0.08	44.1±13.5	98.9±16.0	<0.05	44.1±15.4	94.5±22.8	0.007
Ox (mg)	37.8±15.1	36.3±17.3	0.9	42.5±14.5	42.4±16.8	0.50	40.2±14.2	39.3±16.4	0.80
UA (mg)	583±260	586±228	0.65	712±161	742±157	0.50	648±215	664±202	0.96
SS UA	0.19±0.13	0.5±0.36	<0.05	0.76±0.62	1.38±0.89	<0.05	0.47±0.52	0.94±0.79	0.005
SS CaP	0.74±0.2	0.55±0.33	0.5	1.66±1.04	1.03±0.80	0.14	1.20±0.86	0.79±0.63	0.09
SS CaOx	3.18±0.4	3.58±0.63	0.22	7.14±3.59	8.17±4.99	0.69	5.16±3.18	5.88±4.13	0.33

SS = supersaturation, CaOx = calcium oxalate, CaP = calcium phosphate, UA = uric acid; P values by Wilcoxon signed-rank test; *: effect in hypercalciuric participants different than effect in normal controls; P<0.05 by Mann-Whitney U test.

There was no evidence to suggest the development of metabolic acidosis based on the pre- and post-STS serum bicarbonate levels in the 3 stone forming patients from whom these data were collected (Table 2). All 3 hypercalciuric stone formers who had serum bicarbonate concentration measured after STS experienced changes in urine chemistry consistent with greater metabolic acid loads.

**Table 2:** Serum bicarbonate levels pre and post STS in hypercalciuric stone formers.

	Pre-STS (meq/L)	Post STS (meq/L)
Patient 1	32	28
Patient 2	29	31
Patient 3	24	22
Mean±SD	28.3±4.0	27.0±4.6

Urine volume, creatinine, urea, and sodium excretion remained unchanged in both arms of the study pre- and post-STS, and in the combined data, confirming replication of diets over the course of the collections. Administration of the drug was well tolerated with no patients stopping therapy due to adverse events. All patients were questioned about their experience with the drug: 2 patients had transient episodes of loose or watery stools which resolved despite continued STS administration and 2 patients described foul smelling stool. No deviations from the protocol occurred.

## Discussion

In the first study to examine a full set of urinary analytes after administration of oral STS, neither healthy controls, nor hypercalciuric calcium stone formers, experienced statistically significant increases in urine calcium excretion or calcium oxalate or calcium phosphate supersaturation. In stone-formers, there were decreases in urine pH and citrate, and increases in ammonium excretion without a change in serum [HCO_3_]. Similar results occurred in the control non-stone-formers, with the exception that citrate did not fall. These effects on urine chemistry, with no net change in lithogenicity, would not explain remarkable reductions in stone activity reported in a non-controlled, non-randomized clinical study [6]. Of note, that study also demonstrated no significant difference in calciuria pre- and post-STS.

Our urine results are also not consistent with the findings of Yatzidis et al [6]. In that paper, participants receiving the same 20 mmol dose of STS that we studied excreted about 4 mmol/d of thiosulfate, with only 0.5 mmol/d of sulfate excretion, an unlikely, below-normal, value. We note that we used a similar barium-precipitation method for sulfate measurement and therefore cannot account for the peculiarly low sulfate values reported in that paper. In contrast, we noted less than 1 mmol/d of thiosulfate in the urine of our study participants, and found that excreted sulfate was approximately equimolar with the amount of thiosulfate administered. Similarly, in the rat study, only 6% of orally administered thiosulfate appeared in the urine, considerably less than the excreted sulfate [5].

The present study is consistent with thiosulfate presenting a net acid load, suggested by increases in urine ammonium excretion, and decreases in citrate excretion (in stone-formers) and urine pH. The etiology of this acid load is not clear. Stool losses of bicarbonate or potential base could be an explanation, though most patients had no diarrhea and those who did, experienced it only transiently. Previous reports of metabolic acidosis after parenteral administration of STS for cyanide poisoning and calcific uremic arteriolopathy exist [11], [12]; parenteral administration of STS is not associated with diarrhea. Although only 3 patients had serum [HCO_3_] measurements while taking STS, all 3 had clear evidence of having received an acid load based on urine chemistry while maintaining normal serum [HCO_3_]. The lack of change in serum [HCO_3_] in the stone formers and the lack of change in urine citrate in the controls (in whom serum [HCO_3_] was not measured) suggest that ammoniagenesis was adequate to compensate for the presumed acid load. The difference in citrate excretion between controls and stone-formers despite similar increases in urine ammonium excretion, is interesting but not explained and suggests that perhaps stone-formers have greater renal citrate reabsorption in response to acid loads, contributing to relative hypocitraturia. The decrease in urine pH caused a significant increase in supersaturation of uric acid and could be associated with an increase in uric acid stone formation in some susceptible patients.

Some recent literature has claimed that sodium thiosulfate is a strong acid [13], when the molecule clearly lacks protons to donate. Others suggest that dissolution of sodium thiosulfate leads to formation of thiosulfuric acid [11], which cannot occur given that the pK_a_’s of that strong acid are 0.6 and 1.7 and the common knowledge that the sodium salt of a strong acid is not itself an acid. More likely, the acid load is due to the oxidation of thiosulfate to sulfate by the liver, producing protons [14]. An alternative explanation would be the oxidation of thiosulfate to sulfate by intestinal bacteria with absorption of sulfate and protons by the intestine [15]. Either of these mechanisms is consistent with our finding that oral thiosulfate administration was followed by excretion of sulfate, not thiosulfate, in the urine. However, the possibility that this conversion occurs as the result of metabolism by intestinal bacteria remains open, since intravenously administered thiosulfate is rapidly excreted with only a small portion being metabolized [16]. On the other hand the occurrence of acidosis in dialysis patients treated with parenteral STS for calcific uremic arteriolopathy might be attributable to these reactions occurring endogenously at a slow rate but one sufficient to produce acidosis when excretion is limited by low glomerular filtration rate.

The urine findings are similar to those noted in GHS rats given STS [5]. These animals generally form calcium phosphate stones if fed normal rat chow. STS led to an increase in urine calcium excretion as well as increased urine ammonium, and decreased pH and citrate excretion. Therefore the significant reduction in stone formation was not explained by measurement of conventional urine chemistry and calculation of calcium phosphate or calcium oxalate supersaturations. Though acidification of urine could be expected to reduce formation of calcium phosphate stones, this possibility was discounted because in previous studies, administration of the acidifying agent ammonium chloride was not associated with reduction in stone formation [17]. Increased urine calcium in the rats could be attributable to increased urine sodium excretion, given the sodium content of STS, or due to the metabolic acid load. Neither a significant increase in sodium nor calcium excretion occurred in our participants. In another rat study, ethylene glycol and ammonium chloride-induced nephrocalcinosis was not benefitted by administration of STS. [18].

One limitation of the study is the small sample size of the 2 groups and that the groups are not matched for age. Since the primary outcome was not a comparison of the effects of the drug on the two populations we do not consider the age difference to be important. In fact, combining the results of the 2 groups demonstrated that the results were highly consistent between them with the only difference being a fall in urine citrate in hypercalciuric stone-formers but not in the non-stone-forming controls. Finding healthy people willing to take an investigational drug and do multiple urine collections among a hospital population was more difficult than finding stone formers interested in advancing the knowledge of their disorder and its potential treatment. Only after the results in the healthy group did we realize that it would be desirable to measure serum chemistry after drug administration and because of logistical issues could not get blood while participants were still taking the drug in 2 of the 5 stone formers. Since serum [HCO_3_] did not change in the 3 in whom it was measured despite their significant changes in urine chemistry, we do not believe this small sample is an important limitation to knowing whether an acid load was presented. The urine findings clearly show that it occurred. We cannot speculate regarding the generalizability of these findings to a broader population of calcium stone formers with these or other urinary risk factors for stone formation.

The absence of a significant change in serum [HCO_3_] does not necessarily confirm the safety of long-term STS administration, as the finding is potentially related to two limitations of the study: its small sample size and relatively short duration of STS administration. STS therapy for the prevention of nephrolithiasis would likely need to continue for many years, and as such, studies of at least months to years would be necessary to sufficiently rule out development of sustained metabolic acidosis. A larger study with a longer duration more similar in scale and length to that of Yatzidis [6] would be necessary for a more concrete assessment of the safety of the drug. Yatzidis however did not report any serum or urine variables that would allow determination of whether there was evidence for the metabolic acidosis we show here. The implication of long-standing metabolic acidosis might be to reduce BMD. Despite the lack of an increase in urine calcium excretion in this and previous studies, the long-term effects of protein ingestion and net acid loads to reduce BMD are well known [19]. Patients with hypercalciuria are particularly susceptible to these effects and have lower BMD compared with age- and gender-matched controls [20]. Treatment with STS reduced the load needed to fracture femurs of nonuremic rats when compared to untreated animals; those animals also experienced hypercalciuria [21].

The appropriate therapy for prevention of recurrent calcium phosphate stones is unclear as no randomized controlled trials with this outcome have been completed [22]. The role of administration of citrate is controversial because its effect to alkalinize the urine and increase calcium phosphate supersaturation may, to an extent, negate the effect of alkali to reduce calcium excretion and increased urine citrate to inhibit calcium salt crystallization [23]. Therefore it is worth considering whether an agent that leads to urinary acidification without significant acidosis would be effective for such stones. STS did inhibit calcium phosphate stone formation in GHS rats despite increasing urine calcium, a finding not observed in these human studies [5]. It might therefore be worthwhile to study the effects of STS specifically in this setting. Whether administering it with bisphosphonates or thiazides to diminish potential loss of BMD and reduce calciuria [24], would improve its safety profile and enhance efficacy can only be determined by appropriate clinical trials. However, overall, given the acid-loading effects of STS, as evidenced by the changes in urine chemistry, and the effects on bone characteristics demonstrated in animal studies, we are not confident that STS would be a safe and effective therapy for prevention of recurrent calcium oxalate or calcium phosphate stones.
